# Application of specialized pro‐resolving mediators in periodontitis and peri‐implantitis: a review

**DOI:** 10.1111/eos.12759

**Published:** 2021-02-09

**Authors:** Muhanad Ali, Fang Yang, Adelina S. Plachokova, John A. Jansen, X. Frank Walboomers

**Affiliations:** ^1^ Department of Dentistry, Regenerative Biomaterials Radboud University Medical Center Nijmegen The Netherlands; ^2^ Department of Dentistry, Implantology and Periodontology Radboud University Medical Center Nijmegen The Netherlands

**Keywords:** immune response, lipoxin, peri‐implantitis, periodontitis, specialized pro‐resolving mediators

## Abstract

Scaling and root planning is a key element in the mechanical therapy used for the eradication of biofilm, which is the major etiological factor for periodontitis and peri‐implantitis. However, periodontitis is also a host mediated disease, therefore, removal of the biofilm without adjunctive therapy may not achieve the desired clinical outcome due to persistent activation of the innate and adaptive immune cells. Most recently, even the resident cells of the periodontium, including periodontal ligament fibroblasts, have been shown to produce several inflammatory factors in response to bacterial challenge. With increased understanding of the pathophysiology of periodontitis, more research is focusing on opposing excessive inflammation with specialized pro‐resolving mediators (SPMs). This review article covers the major limitations of current standards of care for periodontitis and peri‐implantitis, and it highlights recent advances and prospects of SPMs in the context of tissue reconstruction and regeneration. Here, we focus primarily on the role of SPMs in restoring tissue homeostasis after periodontal infection.

## INTRODUCTION

Inflammation is central to the pathology of periodontitis and peri‐implantitis following colonization with Gram‐negative bacteria contained in the dental plaque biofilm, such as *Porphyromonas gingivalis* and *Fusobacterium nucleatum* [1]. The inflammatory response is characterized by the recruitment of leukocytes, vascular dilation, increased blood flow, and enhanced permeability of blood capillaries. Typically, the innate immune system induces an inflammatory response capable of eradicating bacteria and returning tissue homeostasis. Failure of the innate immune system to eliminate inflammation and clear immune cells can lead to chronic inflammation, which is characterized by a prolonged and persistent inflammatory response to external stimuli, like bacterial components such lipopolysaccharide (LPS) [2]. During chronic inflammation, inflammatory mediators, such as cytokines, chemokines, and prostaglandins, amplify the inflammatory response by recruiting more leukocytes and platelets to the site of infection [3, 4]. Elevated levels of inflammatory mediators, such as prostaglandin E_2_ (PGE2), closely correlate with the disease severity in patients with periodontitis and peri‐implantitis [5]. Therefore, the primary objective of periodontal therapy is the regulation of the host defense mechanisms underlying chronic inflammation.

Current non‐surgical treatment strategies for periodontitis and peri‐implantitis patients present several challenges, including inability to control inflammation, high cost, and increasing threat of antibacterial resistance [6]. Moreover, traditional management of these diseases has not changed appropriately over the last decades despite an increased understanding of their pathophysiology [7]. Typically, treatment consists of scaling and root planning (SRP) and in ‘refractory cases’ (i.e., cases that do not respond to treatment) or ‘aggressive’ forms (i.e., fast progressive cases with severe damage) of the disease, adjunctive therapy is commonly used, such as the administration of broad‐spectrum antibiotics (e.g., amoxicillin and metronidazole or doxycycline) [8]. However, repetitive use of antibiotics can lead to bacterial resistance and notable side effects, such as nausea, diarrhea, and hypertension [9]. Furthermore, glucocorticoids, such as dexamethasone, are commonly prescribed to manage inflammation associated with chronic inflammatory diseases due to their immunosuppressive actions on the immune system, such as the inhibition of interleukin (IL)‐6, 8 and tumor necrosis factor α (TNFα) [10]. Glucocorticoids are, however, limited by their anti‐proliferative actions on the periodontium, resulting in attachment loss, disruption of transseptal fibers, and down‐migration of the epithelium, as well as bone loss, which can negatively impact implant osseointegration [11]. Nonsteroidal anti‐inflammatory drugs (NSAIDs; e.g., ibuprofen, aspirin, flurbiprofen, indomethacin, naproxen) are widely prescribed in dentistry to reduce inflammation and manage pain associated with invasive dental procedures. NSAIDSs work by inhibiting cyclooxygenase (COX) enzymes and thereby limit synthesis of prostaglandins, such as PGE2 and PGI2 [12, 13]. NSAIDs can produce several beneficial effects in periodontitis and peri‐implantitis patients, including periodontal and implant wound healing [14]. However, NSAIDs can take a long time to produce a therapeutic response (years rather than months) and are associated with undesirable effects that include gastrointestinal problems and hemorrhage (due to decreased platelet aggregation), as well as renal and hepatic dysfunction [12]. Additionally, patients on long‐term NSAIDs also run the risk of accelerated bone loss if they cease taking them. Hence, conventional periodontal therapy can be insufficient for preventing disease progression and eliminating subgingival infection without eliciting adverse side effects.

Alternatively, specialized pro‐resolving mediators (SPMs) are endogenous cell‐signaling molecules released during inflammation to promote resolution of inflammation, which is a coordinated response aimed at restoring tissue integrity and function [15, 16, 17, 18]. Despite the cellular and molecular mechanisms of inflammation being extensively described in the literature for periodontitis and peri‐implantitis [19, 20, 21], a comprehensive review on the effect of SPMs on immune cells (innate and adaptive) and periodontal cells is lacking. In this review, we consider the use of SPMs as a new therapeutic strategy to replace antibiotics and mitigate the growing problem of antibiotic resistance. Here, the function of SPMs is addressed, with a focus on how SPMs affect the innate and adaptive immune system cells. Finally, we highlight recent discoveries regarding the anti‐inflammatory effects of SPMs in harnessing endogenous resolution responses in periodontal cells.

## MATERIAL AND METHODS

This narrative review is based on preclinical and clinical studies in English without restrictions regarding year of publication. The review focused on the following questions:

- What is the role of inflammation in periodontal and peri‐implant tissue breakdown?
- Which cell types of the innate and adaptive immune system (as well as cells of the periodontium) are involved in mediating periodontal tissue destruction?
- What is the therapeutic application of SPMs in periodontal tissue regeneration?

The electronic databases Medline (via PubMed), Scopus, and Embase were searched for eligible studies in the field of periodontology, implantology, and immunology. The main search terms were ‘pro‐resolving agents,’ ‘periodontitis,’ ‘peri‐implantitis,’ ‘resolution,’ and ‘inflammation.’ Subsequently, the title and abstract of obtained articles were screened for relevancy to the research criteria. The inclusion criteria consisted of the following: (i) full text original studies in the English language; (ii) research articles demonstrating the effect(s) of SPMs in in vitro and in vivo model systems; and (iii) experimental studies involving the clinical periodontitis or peri‐implantitis situation. The references contained in original articles and review articles were also searched for possible inclusion. No further inclusion criteria were applied.

### Pathogenesis of periodontitis and peri‐implantitis

#### Periodontitis

Periodontitis is characterized by bacterial infection leading to inflammation of the periodontium, which makes up the specialized tissues that surround and support the teeth [1]. Periodontitis develops from a superficial reversible type of inflammation, gingivitis, that develops in the gingiva (or gums). In gingivitis, regeneration processes are activated in such a way that complete replacement with new cells usually occurs without any permanent tissue damage [6]. In periodontitis, however, healing is overridden by irreversible scarring, fibrosis, and destruction of the cellular matrix, which usually requires lifelong treatment [2]. Inflammation is central to the pathology of diseases of the periodontium and a thorough understanding is key to the development of novel treatment strategies that can promote wound healing and restore tissue function.

#### Peri‐implantitis

Dental implants were first introduced in the 1950s, and since the 1980s they have become increasingly popular for replacing missing teeth and enhancing masticatory function [22]. The prevalence of dental implants in the United States is estimated to increase from 5.7% to 17% per year by 2026 [23]. With increasing reliance on dental implants, complications and dental implant failures have become increasingly problematic due to the aging population and increasing cost of treatment [24]. The clinical signs of peri‐implantitis consist of bleeding on probing (BOP), redness, edema, and bone loss [22]. There are several risk factors for dental implant failures, including the patient's medical history (e.g., diabetes, periodontitis, and other systemic disease), lifestyle (e.g., smoking, and alcohol consumption), and implant surface characteristics (e.g., smooth or rough) [22]. For instance, Berglundh et al. [25] showed that implants with a rough surface morphology elicited more bone loss compared with smooth‐surfaced implants. Furthermore, excessive biomechanical forces can lead to microfractures in the bone around the implants, which can result in implant failure [26]. Still, a main reason for dental implant failure is the consistent exposure to the oral microflora, which includes oral bacteria that can easily colonize the dental implant surface and form a pathogenic biofilm [27]. A pathogenic biofilm can result in mucosal inflammation of the soft tissue (or peri‐implant mucositis), alveolar bone resorption, and eventually implant loss over time [22]. Although both periodontitis and peri‐implantitis have similar pathologies, there are key histopathological differences that discern them.

### Histopathological differences in periodontitis and peri‐implantitis

**FIGURE 1:**
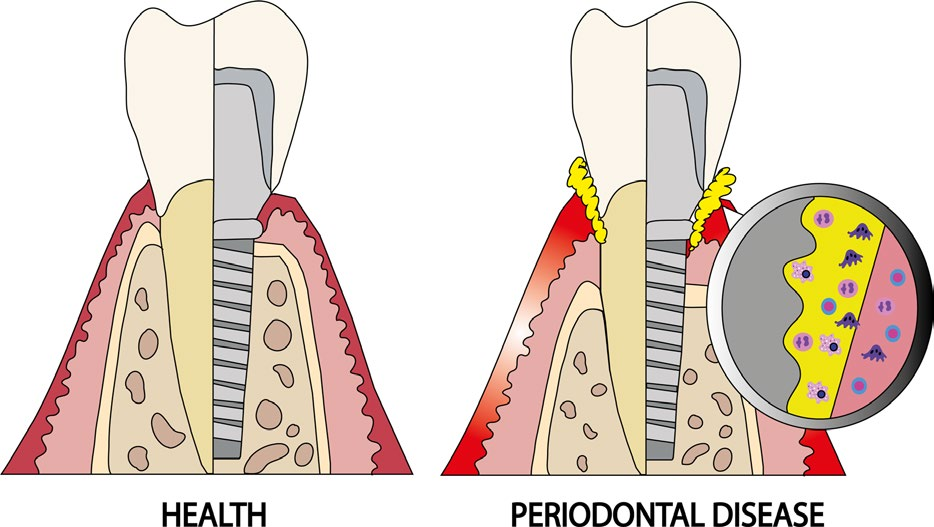
Periodontal and peri‐implant tissues in health (left) and disease (right). In the diseased conditions, a biofilm (in yellow) develops on the tooth or implant surface resulting in inflammation and destruction of the supporting tissues. Unresolved inflammation leads to deep periodontal/peri‐implant pockets and an increase in immune cell infiltration at the inflammatory lesion.

Periodontitis and peri‐implantitis are results of unresolved inflammation occurring in response to the pathogenic bacterial biofilm residing on the tooth (or implant) surface (Figure 1) [19]. Despite both conditions being inflammatory in nature, results from experimental animal studies indicate key histopathological differences that could explain variations in disease onset and progression [28]. Earlier experiments in large animals, such as dogs and monkeys, have identified differences between these two inflammatory lesions [29, 30, 31]. In these studies, experimental periodontitis and peri‐implantitis were established by placing ligatures around the neck of the tooth (or implant), which allowed subgingival biofilm formation. Characterization of human biopsy samples revealed several quantitative immunological differences between periodontitis and peri‐implantitis lesions [19]. For instance, histological analysis of peri‐implantitis lesions showed a more pronounced inflammatory cell infiltration compared to periodontitis lesions [32, 33]. Bullon et al. [34] and Gualini & Berglundh [21] reported higher levels of macrophages, plasma cells, lymphocytes, T cells, and B cells in human periodontitis and peri‐implantitis lesions compared with healthy tissues. Moreover, the levels of T cells were significantly higher in human peri‐implantitis patients compared with periodontitis patients, and this has been shown to mediate IL‐17‐induced osteoclastogenesis and bone resorption via nuclear factor‐κB ligand (RANKL) activation [35, 36]. Interestingly, Konttinen et al. [37] showed that peri‐implantitis lesions contained more cells positive for IL‐1α, IL‐6, and TNF‐α compared with periodontitis lesions. Generally, it was found that peri‐implantitis lesions contained a higher number of cells positive for pro‐inflammatory cytokines and chemokines (IL‐1β, IL‐6) than periodontitis lesions and healthy tissues [38].

Furthermore, histological examination of the two lesions showed that tissue destruction and bone loss was more pronounced surrounding implants compared with teeth [30, 39, 40]. Additionally, peri‐implantitis lesions were larger, extended closer to the alveolar bone crest, and contained more osteoclasts compared to periodontitis lesions [20]. The more pronounced tissue destruction observed in peri‐implantitis lesions is attributed to a combination of biomechanical overload and the presence of more pathogenic bacteria [26]. Additionally, the differences in the collagen fiber orientation (parallel to implant and perpendicular with teeth) and a larger vasculature density may explain the faster pattern of destruction with peri‐implantitis than with periodontitis lesions [28]. Overall, there are quantitative histopathological differences between the two lesions, and these differences have indicated a more pronounced inflammatory response in peri‐implantitis compared with periodontitis lesions.

### Specialized pro‐resolving mediators resolve inflammation

Chronic unresolved inflammation in periodontal and peri‐implant disease can lead to irreversible tissue destruction. Over the past decade, there has been increasing interest in using SPMs for the resolution of inflammation for several inflammatory diseases, including periodontitis and peri‐implantitis. Recent studies have confirmed that resolution of inflammation is an active process rather than a passive one, and this process is normally maintained by endogenous pro‐resolution mediators (or agonists) [41]. The discovery of SPMs, such as lipoxins, resolvins, and protectins, have created new possibilities for development of therapies targeting resolution pathways to terminate inflammation and facilitate the return of inflamed tissues to homeostasis. Resolution of inflammation is a coordinated process that provides ‘stop signals’ to terminate and eliminate infiltration of immune cells to the inflammation site. SPMs are readily generated by cells to limit leukocyte recruitment, chemotaxis to inflamed sites, and the progression of acute inflammation to chronic inflammation. Additionally, SPMs promote the clearance of leukocytes, fibrin, and exudates, which restore damaged tissue function. In general, SPMs work by activating repair mechanisms and returning inflamed (or diseased) tissue back to normal physiological function (or homeostasis). However, relatively little is known regarding the effect of SPMs on the innate and adaptive immune cells, as well as on cells of the periodontium.

### Cellular targets for specialized pro‐resolving mediators

SPMs limit inflammation by utilizing pro‐resolution pathways to promote resolution of inflammation and return tissue homeostasis (Table 1). SPMs induce effects similar to those of glucocorticoids and NSAIDs without producing notable side effects, such as liver and kidney damage [16]. The following sections will focus on the molecular background behind the therapeutic mechanisms involved in the termination of inflammation by SPMs. Additionally, the properties and function of different immune (acute and adaptive) and periodontium cells in tissue inflammation and bone resorption will be discussed in the context of periodontitis and peri‐implantitis. Finally, the effect of SPMs on inflammation will be discussed for each cell type.

**TABLE 1:** Summary of the main effects and concentration of specialized pro‐resolving mediators.

SPM	Concentration of SPM	Cell/animal type	Main effect mediated by SPM	Ref.
LXA4	In vitro: 500 nM	Human leukocytes	Inhibited LPS‐stimulated peroxynitrite formation, NF‐κB and AP−1 activation, and IL−8 gene expression	[15]
LXA4	In vitro: 25−100 nM In vivo: 10 μg/kg	Murine RAW264.7 cells Mice	Suppressed osteoclastogenesis and prevented ovariectomy‐induced bone loss	[16]
LXA4	In vitro: 1−100 nM	Human stem cells of the apical papilla	Enhanced proliferation, migration, and promoted wound healing capacity; inhibited secretion of cytokines and chemokines in a dose‐dependent manner, including IL−6, IL−8, CXCL10, CCL2, CCL11, VEGF	[17]
LXA4	In vitro: 10−100 nM	Murine RAW264.7 and MC3 T3‐E1 osteoblasts co‐culture	Inhibited secretion of pro‐inflammatory cytokines, including TNFα, IL−1β, PGE2 and GM‐CSF	[18]
LXA4	In vitro: 30–2000 nM In vivo: 200 mg/kg	Human neutrophils Mice	Enhanced resolution of inflammation by overriding anti‐apoptosis signals	[50]
LXA4	In vitro: 100–1000 nM	Human & murine macrophages	Impaired apoptosis signaling in macrophages via activation of PI3 K‐Akt signaling	[54]
LXA4	In vitro: 10 nM	Human monocytes and polymorphonuclear neutrophil (PMN)	Promoted phagocytosis of apoptotic PMNs by monocyte derived macrophages	[63]
PD1	In vitro: n/a In vivo: 100 ng per animal	Human PBMC and T Cells Mice	Inhibited T‐cell migration and production of TNFα; promoted apoptosis of T cells	[81]
LXA4	In vitro: 100 nM	Human PMNs	Upregulated the expression of the anti‐inflammatory gene, NAB1	[85]
LXA4	In vitro:100 nM In vivo: 1 μg per mouse	Human peripheral B cells Mice	Inhibited B cell production of antibodies (IgM and IgG) and proliferation	[93]
RvD1	In vitro: 0.1–10 ng/ml	Human periodontal ligament fibroblasts and peripheral blood mononuclear cells	Decreased production of PGE2; decreased cytokine induced production of PGE2; upregulated LXA_4_ production in both cell types	[95]
RvE1	In vivo: 8 μl per animal	Rabbits	Induced complete resolution of inflammation of the soft and hard tissues	[106]
RvD1	In vitro: 1000 nM	Human gingival fibroblasts	Reduced the expression of IL−6 and MCP−1, GRO, and marginally increased the production of TGF‐β1	[110]
RvE1	In vitro: 1–3 ng/ml	Murine osteoclasts	Reduced osteoclast differentiation and bone resorption activity	[121]
RvE1	In vitro: 10 nM	Murine osteoclasts	Reduced osteoclast formation	[122]
RvD1	In vitro: 100–500 nM In vivo: 500–1000 ng	Human RAW264.7 cells Mice	Prevented bone resorption in vitro and in vivo	[127]
RvE1	In vitro: 100 nM	Murine osteoclasts	Decreased production of RANKL and maintained a RANKL/OPG ratio more favorable for bone formation; significantly decreased osteoclast differentiation and bone resorption	[136]
RvE1	In vitro: 50–200 nM	Murine RAW264.7 cells	Inhibited osteoclatogensis and bone resorption by suppressing IL−17 induced RANKL expression in osteoclasts	[137]

Abbreviations: AP‐1, activator protein 1; CCL, C‐C motif chemokine ligand; CXCL, C‐X‐C motif chemokine ligand 10; GM‐CSF, Granulocyte‐macrophage colony‐stimulating factor; GRO, growth‐regulated oncogene; IFNγ, Interferon gamma; IgG, Immunoglobulin G; IgM, Immunoglobulin M; IL, interleukin; LPS, lipopolysaccharide; LXA4, lipoxin A4; NAB1, NGFI‐A Binding Protein 1; NF‐κB, Nuclear factor kappa B; OPG, osteoprotegerin; PBMC, peripheral blood mononuclear cells; PD1, Protectin D1; PGE2, prostaglandin E_2_; PI3 K‐Akt, phosphatidylinositol‐4, 5‐bisphosphate 3‐kinase; RANKL, Receptor activator of nuclear factor kappa‐Β ligand; RvD1, Resolvin D1; RvE1, MCP‐1, monocyte chemoattractant protein 1; RvE1, Resolvin E1; SPM, Specialized pro‐resolving mediator; TGF‐β1, transforming growth factor‐β1; TNFα, tumor necrosis factor alpha; VEGF, vascular endothelial growth factor.

### Innate immune cells

#### Polymorphonuclear neutrophils

**FIGURE 2:**
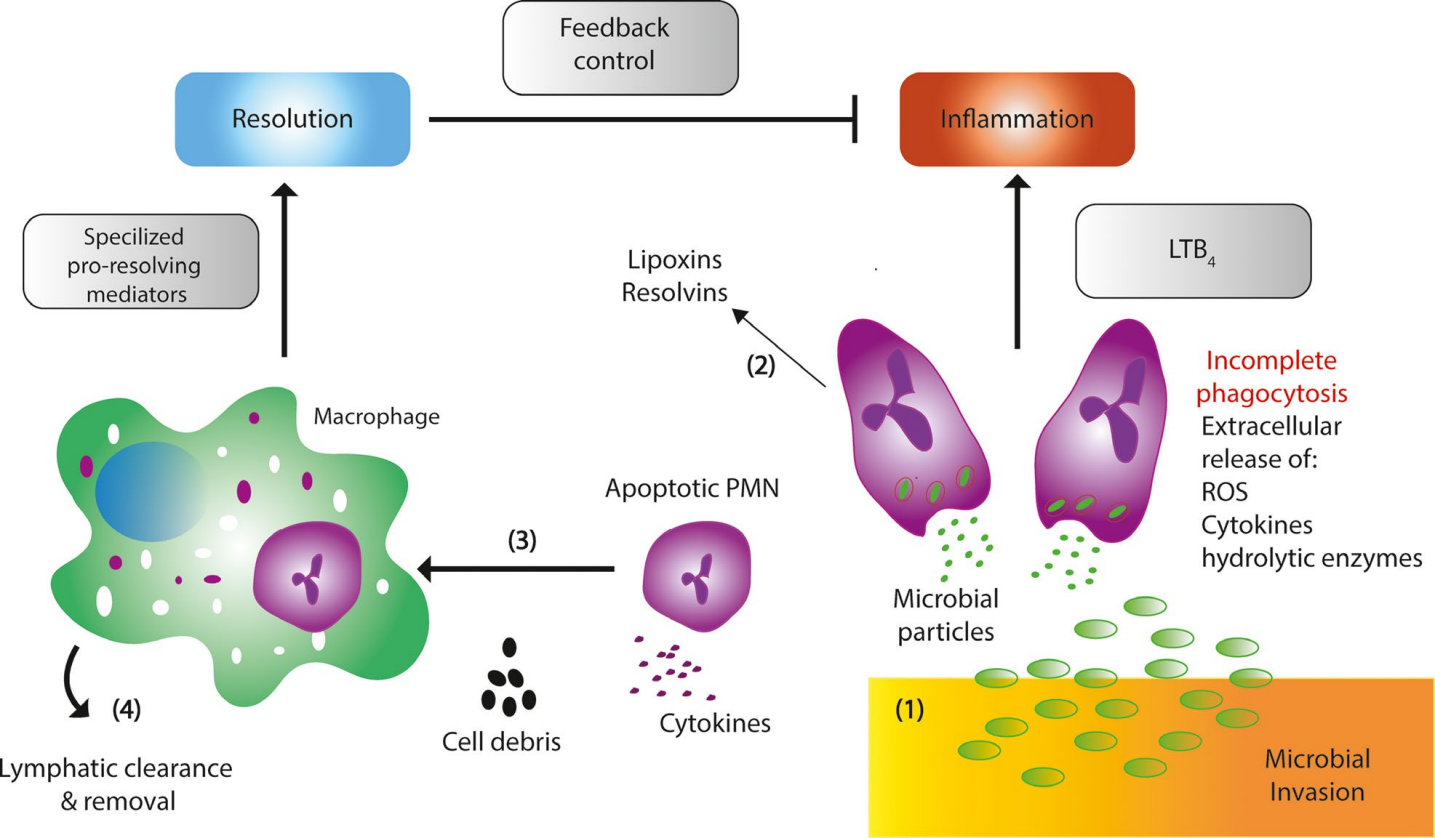
The role of specialized pro‐resolving mediators (SPMs) in the inhibition of polymorphonucleated neutrophils (PMNs). (1) In the lower right of the figure, microbial invasion injury induces chemotactic signals, including leukotriene B4 (LTB4), reactive oxygen species (ROS), cytokines, and hydrolytic enzymes, which activate PMNs chemotaxis to the site of injury. (2) As PMNs accumulate in the inflammation site, cell‐cell interactions mediated transcellular biosynthesis of SPMs, such as lipoxins and resolvins. (3) When resolvins and protectins are synthesized, they start to stimulate phagocytosis of apoptotic PMNs and cell debris by macrophages. (4) SPMs enhance macrophage uptake and the clearance and synthesis of lipoxins and resolvins, which inhibit inflammation through feedback mechanisms to resolve inflammation and return to tissue to homeostasis from local tissue injury.

Polymorphonuclear neutrophils (PMNs) are the most abundant white blood cells and the first line of defense recruited by the innate immune system in response to bacterial invasion (Figure 2) [42, 43]. During infection, circulating PMNs in the blood stream respond to chemoattractants (e.g., cytokines, chemokines, and leukotrienes) and adhesion molecules (e.g., selectins, integrins) to accumulate at the site of inflammation. PMNs activate several anti‐microbial functions, including phagocytosis, the release of soluble anti‐microbials, and the generation of neutrophil extracellular nets and myeloperoxidase. Myeloperoxidase secreted from the azurophil granules of PMNs serves two main functions: (i) eradicate pathogens by forming highly reactive halide‐derived oxidants, and (ii) prolong the life span of PMNs by promoting powerful anti‐apoptotic signals [44]. Delayed PMN clearance is a key factor that promotes tissue destruction in several inflammatory diseases, including acute respiratory syndrome, certain types of cancer, and periodontal disease [45, 46]. Hence, the clearance of PMNs from the site of inflammation by phagocytes (e.g., monocytes and macrophages) is crucial to limiting inflammation and, therefore, preventing tissue damage [47].

Since PMNs have been implicated in inflammatory diseases, controlling PMN infiltration has been a major pharmaceutical target for treating inflammation‐induced tissue destruction. To assess the effect of SPMs on PMN infiltration, fluorometric measurements of cytosolic calcium (Ca^2+^) were carried out in vitro using human PMNs obtained from healthy volunteers [48]. In that study, lipoxin intermediate (leukotriene A4) was shown to promote migration of PMNs by increasing cytosolic Ca^2+^ levels via ALX/FPR2 receptor‐mediated interactions. Higher cytosolic Ca^2+^ concentrations correlated with increased transendothelial migration of PMNs from assembly of cytoskeletal elements into pseudopods, leading to the clearance of PMNs from the inflammation site [49]. In addition to promoting clearance, lipoxin induced the activation of apoptotic signals in PMNs by suppressing apoptosis‐promoting proteins, such as serum amyloid A [50]. Moreover, studies have shown that lipoxin prevents tissue injury by inhibiting the formation of reactive nitrogen intermediates, such as peroxynitrite (ONOO) [51, 52]. In addition, lipoxin has been shown to inhibit the accumulation of nuclear factor κB (NF‐κB) and activator protein‐1 (AP‐1), and this decreased the production of the inflammatory cytokines that normally promote PMN accumulation and activation (e.g., IL‐8). Furthermore, resolvins have also been shown to inhibit PMN migration, infiltration, and accumulation at the inflammation site [48, 53]. To this end, inhibition of PMN recruitment by SPMs is vital for preventing the progression of inflammation.

#### Macrophages

Macrophages contribute to the clearance of pathogens and cellular debris from the inflammation site [54]. Macrophages can be polarized into two phenotypes depending on their microenvironment: the pro‐inflammatory (M1) or the anti‐inflammatory (M2) subtypes. M1‐type macrophage polarization occurs in response to production of pro‐inflammatory cytokines, such interferon gamma (IFN‐γ), or by interactions with bacterial LPS. M1‐type macrophages exhibit an inflammatory phenotype, which is associated with higher levels of pro‐inflammatory mediators, such as IL‐1β, IL‐6, TNFα, and nitric oxide (NO) [55]. In addition, M1‐type macrophages contribute to the elimination of pathogenic microorganisms by phagocytosis of pathogenic microorganisms. However, persistent activation of pro‐inflammatory M1 macrophages produces excessive release of the pro‐inflammatory mediators that contribute to chronic inflammation and correlate with tissue injury [56]. In contrast to M1‐macrophages, M2‐primed macrophages secrete anti‐inflammatory cytokines, such as IL‐4 and IL‐3, both of which have several important functions required for the resolution of inflammation and wound‐healing [57]. Among these functions, M2‐primed macrophages also phagocytose apoptotic PMNs and pathogens, which facilitates the return of tissue homeostasis [58]. The clearance of apoptotic PMNs is vital for redirecting polarization of macrophages from the M1 to the M2 phenotype [59]. Hence, macrophage polarization in gingival tissue is an important disease indicator and a critical therapeutic target for controlling disease progression in periodontal inflammation [60].

Lipoxins have been shown to promote resolution of the inflammation in a concentration‐dependent manner by delaying apoptosis of macrophages and enhancing phagocytosis of PMNs [61]. Furthermore, lipoxins have been shown to prolong the survival of macrophages by activating anti‐apoptotic signaling pathways, including phosphatidylinositol‐4, 5‐bisphosphate 3‐kinase (PI3 K‐Akt) and extracellular signal‐regulated kinase (ERK) [54]. The activation of PI3 K‐Akt and ERK signaling pathways increases the production of anti‐apoptotic mediators, such as myeloid cell leukemia 1 (Mcl‐1), and leads to the suppression of pro‐apoptotic mediators, such as caspases, which inhibit apoptosis in macrophages [54, 62]. In general, SPMs can prevent excessive inflammation by promoting macrophage survival and phagocytosis of inflammatory cells and pathogens at the inflammation site [63, 64].

### Adaptive immune cells

#### T cells

**FIGURE 3:**
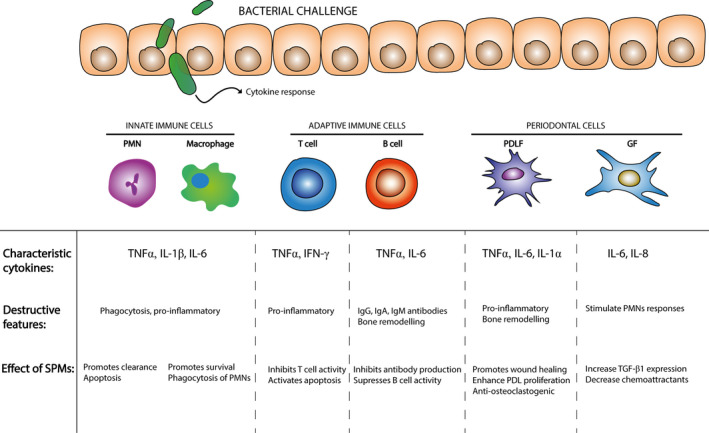
Cytokines and periodontal disease. Bacterial challenge induces an inflammatory cytokine response. The innate immune system induces activation of PMNs and macrophages leading to a pro‐inflammatory immune response. Moreover, the adaptive immune system contributes to polarization and activation of different subsets of T cells, including T‐helper 2 cells, which are associated with inflammatory bone destruction in periodontitis and peri‐implantitis. Periodontal based cells (PDLFs and GFs) are mostly responsible for inflammation and bone remodeling. GF, gingival fibroblasts; PDLF, periodontal ligament fibroblasts; PMN, polymorphonuclear neutrophils.

In addition to the innate immune system, adaptive immune cells also play a prominent role in orchestrating periodontal tissue damage (Figure 3). T‐helper cells are activated and polarized into two distinct subtypes: T‐helper 1 (Th1) or T‐helper 2 (Th2) [65, 66]. Th1 cells are associated with pro‐inflammatory processes and bone destruction, while Th2 cells are more related to anti‐inflammatory and protective effects [67]. Several inflammatory chronic diseases are associated with an imbalance of Th1/Th2 responses, including periodontal disease [68, 69, 70]. Th1 type responses are promoted by the production of inflammatory mediators (mainly IL‐12 and IFN‐γ) by antigen‐presenting cells (such as dendritic cells), leading to osteoclastogenesis and bone loss in vivo [71, 72]. Previous studies have shown that high IFN‐γ levels in human and experimental periodontitis lesions correlate with increased disease severity [73, 74, 75]. In addition to the Th1 and Th2 T‐cell subtypes, Th17 cells are a distinct subset of T cells involved in autoimmune and inflammatory processes, and they are characterized by IL‐17 production [76]. Regulatory T cells (Tregs) are another T‐cell subtype involved in tolerance to self‐antigens and the prevention of autoimmune disease via the production of anti‐inflammatory cytokines, such as transforming growth factor‐β (TGF‐β) [77]. Overall, the Th1 primed T cell subsets play a major role in chronic inflammation and present an attractive therapeutic target for the treatment of chronic inflammatory diseases.

Cell culture studies focusing on the resolution of inflammation tend to focus exclusively on acute conditions mediated by the innate immune system. Therefore, little is known about the actions of SPMs on the cellular components of the adaptive immune system. Besides their role in promoting Th1‐inflammatory responses, anti‐inflammatory dendritic cells can inhibit T‐cell proliferation via the expression of indoleamine 2,3,‐dioxygenase (IDO) in response to a combination of stimulatory factors, such as PGE2 and TNFα [78]. IDO is an enzyme involved in the catabolism of tryptophan, which produces metabolites that are cytotoxic for effector T cells, such as kynurenine, 3‐hydroxykyrunine, and 3‐hydroxy‐anthranilic acid [79]. Lipoxin has been shown to stimulate IDO expression in murine bone marrow‐derived dendritic cells in vitro [80, 81]. Additionally, Resolvin E1 (RvE1) has been shown to inhibit IL‐12 production in human microbially‐activated dendritic cells via interactions with ChemR23 receptors in vitro [82]. Dendritic cells exposed to RvE1 have been shown to differentiate into a tolerogenic subset, which is characterized by low expression levels of IL‐12 and a high expression of IL‐10 in response to bacterial LPS stimulation [80]. Furthermore, lipoxin has been shown to promote the resolution of inflammation by stimulating several apoptotic signaling pathways in activated CD4^+^ T cells, such as IL‐1R‐associated kinase (IRAK‐M), IκB kinases (IKK), or suppressor of cytokine signaling (SOCS) [80, 81, 83]. In addition, lipoxin has been shown to inhibit the production of pro‐inflammatory cytokines expression (IL‐2 and TNFα) by inhibiting the early growth response 1 gene (EGR1) and the transcriptional corepressor NGFI‐A binding protein 1 (NAB1) expression in stimulated T cells [84, 85].

#### B cells

B cells appear to play a prominent role in the onset and progression of chronic inflammatory and autoimmune disease [86]. B cells are involved in the humoral branch of immunity and have been shown to accumulate at the site of oral infection following an immune response by innate immune cells (PMNs and monocytes) [87]. In progressive periodontitis lesions, B cells are phenotypically characterized by the secretion of immunoglobulin G (IgG), IgA, and IgM antibodies, which are specific to periodontal bacteria (e.g., *P. gingivalis*) and one of the prominent pathological features of periodontitis [88]. Moreover, primed B cells have been shown to promote periodontal bone loss via the expression of several osteoclastogenic factors, such as TNFα and IL‐6 [89]. Furthermore, of the total amount of leukocytes that resides in periodontitis lesions, plasma cells accounted for 50% of cells and B cells accounted for approximately 20% [90, 91]. More importantly, the proportion of B cells in periodontal lesions positively correlates with disease severity [92]. Due to their involvement in severe periodontal disease, B cells present a potential therapeutic target for the treatment of periodontitis and peri‐implantitis.

Lipoxin type A4 (LXA4) has been shown to inhibit the activity of memory B cell proliferation and the production of antibodies. Moreover, Ramon et al. [93] showed that LXA4 suppressed IgM and IgG antibodies in activated B cells in a concentration‐dependent manner. In addition, LXA4 decreased NF‐κB translocation to the nucleus, resulting in the suppression of B cell activation, proliferation, and differentiation in vitro [93]. The inhibition of antibody production by memory B cells is crucial for limiting inflammation and building long‐term protection against infiltrating pathogens.

### Periodontal cells

#### Periodontal ligament fibroblasts

Periodontal ligament fibroblasts (PDLFs) are connective tissue cells that originate from ectomesenchyme tissue of the dental follicle and reside in the periodontal ligament (PDL). PDLFs are responsible for connecting the teeth to the alveolar bone and are characterized by their spindle‐shaped and elongated morphological appearance [94]. PDLFs are a specialized subset of fibroblasts responsible for collagen production (e.g., type V collagen), wound healing, and tissue repair [95]. In addition to these activities, PDLFs play a crucial role in inflammation and alveolar bone remodeling. PDLFs promote the expression of pro‐inflammatory cytokines and chemokines (e.g., IL‐1α, IL‐6, and TNFα) in response to the infiltration of microorganisms (e.g., *P. gingivalis*) or microbial components (e.g., LPS) leading to tissue‐destructive responses [96, 97]. Toll‐like receptors (TLRs) play an important role in triggering the activation of intracellular signaling cascades in response to microbial infections. The activation of toll‐like receptor 4 (TLR4) in response to bacterial stimulation results in the secretion of pro‐inflammatory cytokines and chemokines via the activation of the MyD88/NF‐κB intracellular signaling pathway [98]. Among the cytokines released during inflammation, IL‐1α is an important regulatory mediator of bone demineralization and connective tissue matrix destruction [99]. Persistent production of inflammatory cytokines and chemokines promote the production of secondary inflammatory mediators (e.g., IL‐8, macrophage inflammatory protein‐1α, CCL5, CC17, and CXCL13) that sustain the migration of PMNs, monocytes, plasma, lymphocytes, and mast cells to the inflammation site [100, 101, 102]. In addition, inflammatory mediators produced by PDLFs in response to bacterial stimulation can also modulate bone remodeling by stimulating osteoclast activity [103]. PDLFs express osteoclast stimulating factors (including RANKL, TNFα, and IL‐6) by activating intracellular signaling pathways, such as NF‐κB and mitogen‐activated protein kinases (MAPKs), that are associated with osteoclastogenesis and alveolar bone resorption [102, 104]. To this end, PDLFs are a pharmacological target for the treatment of inflammation‐induced bone resorption in periodontitis and peri‐implantitis [96, 105].

The ability of resolvin E1 (RvE1) to restore lost periodontal tissues was notably demonstrated in a rabbit model of periodontitis by Hasturk et al. [106]. Additionally, Gaudin et al. [17] showed that treatment of periodontitis lesions with LXA4 promoted wound healing and restored tissue homeostasis. LXA4 treatment correlated with the inhibition of LPS‐induced production of pro‐inflammatory mediators in a dose‐dependent manner (e.g., IL‐1β, TNFα, and nitric oxide). Furthermore, Mustafa et al. [95] showed that Resolvin D1 (RvD1) treatment protected the periodontal ligament from chronic inflammation and promoted tissue healing and regeneration in vivo by decreasing PGE2 production. In addition, RvD1 enhanced the proliferation rate of PDLFs and promoted wound closure. Based on these studies, PDLFs present a primary target for SPMs to promote periodontal regeneration and restoration of the periodontal integrity in periodontitis lesions.

#### Gingival fibroblasts

Gingival fibroblasts are connective tissue cells with a spindle‐like appearance located in the gingiva apical to the gingival epithelium [97]. Although PDLFs and gingival fibroblasts are spatially located close to one another, they have distinct functional activities in the repair and remodeling of periodontal tissue and the adjacent alveolar bone [107]. For instance, PDLFs express high levels of alkaline phosphatase (ALP; an indicator of osteoblast functionality) and have a higher capacity for the formation of hard tissues compared with gingival fibroblasts [108, 109]. Although gingival fibroblasts also participate in the inflammatory process, there are distinct differences in gene expression between PDLFs and gingival fibroblasts [107]. Gingival fibroblasts are more responsive to bacterial stimulation compared with PDLFs, resulting in higher expression levels of IL‐6 and IL‐8; this is mainly because they frequently encounter virulence factors based on their location in the gingival epithelium [97]. In contrast, PDLFs are located closer to the alveolar bone compared to other cell types residing in the periodontium and, hence, play a more important role in bone remodeling. Gingival fibroblasts are also commonly associated with a higher expression of genes associated with proliferation and metabolism (e.g., Cdc25B, STK15, and CDC25B), and hence, possess a faster proliferation capacity than PDLFs [107]. Due to their involvement in tissue repair and remodeling, gingival fibroblasts are essential targets for inducing protective effect by SPMs.

Human gingival fibroblasts stimulated with *P. gingivalis* express higher levels of pro‐inflammatory cytokines and strong chemotactic factors for PMNs and monocytes compared to their unstimulated counterparts [110]. Treatment of gingival fibroblasts with RvD1 has been shown to inhibit growth‐regulated oncogene expression‐α (GRO‐α), which is a strong chemoattractant for PMNs. Additionally, RvD1 treatment was also shown to inhibit monocyte chemoattractant protein‐1 (MCP‐1) levels, which is associated with the recruitment of mononuclear inflammatory cells to the site of infection and also positively correlates with disease severity [111]. Furthermore, treatment with RvD1 increased the expression of TGF‐β1, which mediates a wide range of anti‐inflammatory and immunosuppressive activities, including the inhibition of pro‐inflammatory cytokines production (e.g., TNFα, IL‐1, and IL‐12) and the suppression of T cells, B cells, macrophages, and monocytes activity [112, 113]. Animal experiments have demonstrated that TGF‐β1 knockout mice develop a severe inflammatory reaction and die within weeks of birth, suggesting that TGF‐β1 is critical for dampening the inflammatory response and promoting wound healing [114]. These studies suggest that RvD1 has a potential application in wound healing and plays an important role in the resolution of inflammation and regeneration of gingival tissue [110].

#### Osteoclasts

Osteoclasts are specialized bone resorptive cells responsible for the degradation of mineralized bone [115]. Osteoclasts develop from mononucleated osteoclast precursor cells into multinucleated, tartrate‐resistant acid phosphatase (TRAP)‐positive cells in response to stimulatory factors expressed by osteoblasts [116]. Chronic infection by Gram‐negative bacteria can lead to bone destruction due to enhanced osteoclast activity, resulting in alveolar bone resorption [117]. Alveolar bone resorption results from a homeostatic imbalance between bone formation by osteoblasts and bone resorption by osteoclasts during periodontal infection [118]. The destructive effect of osteoclasts is promoted by inflammatory mediators, including macrophage colony‐stimulating factor (M‐CSF), RANKL, TNFα, IL‐1, and PGE2 [119]. A microbial LPS challenge has been shown to activate pathogenic processes involved in bone degradation by increasing the number and activity of TRAP‐positive osteoclasts in vivo [120]. Due to their involvement in bone remodeling, osteoclasts present an important target for the prevention of alveolar bone erosion and, subsequently, tooth (or implant) loss.

Specialized pro‐resolving mediators possess several important functions that can restore lost periodontal tissue, including bone [106]. Their role as therapeutic targets for the treatment of inflammation‐induced bone resorption has previously been demonstrated in several in vitro disease models, including periodontal disease. With respect to bone metabolism, several studies demonstrated the ability of SPMs to prevent bone resorption by inhibiting gene expression. SPMs have been shown to inhibit mRNA expression of osteoclastogenesis promoting transcription factors (i.e., NF‐κB, NFATcl and c‐fos), which normally function to promote osteoclastic differentiation by activating the expression of osteoclast‐specific genes (e.g., TRAP, cathepsin K, and MMP‐9) [16, 121, 122, 123]. For instance, RvE1 has been shown to inhibit the expression of osteoclast‐specific genes by decreasing nuclear translocation of transcription factors from the cytoplasm to the nucleus in osteoclast precursor cells in vitro [124]. In addition, Liu et al. [16] reported that lipoxin inhibited multiple downstream pathways that are responsible for inflammation‐induced bone metabolism, including the RANK signaling pathway in vitro. This was later shown to inhibit osteoclastogenesis and decrease inflammation‐induced bone destruction in vivo [16, 120]. Furthermore, RvD1 was shown to inhibit osteoclast differentiation and bone resorption in vitro, and RANKL/M‐CSF‐induced bone resorption in vivo by acting on RvE1 receptor chemokine‐like receptor 1 (chemR23 or CMKLR) [125, 126, 127]. Concordantly, Van Dyke [128] revealed that topical administration of RvE1 three times per week to rabbits with periodontitis prevented bone loss induced by *P. gingivalis* in vivo. Altogether, these studies show that SPMs can protect periodontal tissue from bacterial‐induced activation of the inflammatory pathways that lead to tissue‐destructive responses.

#### Osteoblasts

Osteoblasts are specialized bone‐forming cells derived from mesenchymal stem cells in the bone marrow stroma responsible for deposition of mineralized bone [118]. During bone formation (or ossification), osteoblasts mineralize secreted bone matrix proteins, including type I collagen and other various components, into hard tissue composed of hydroxyapatite (or bone) [129, 130]. Besides their role in bone remodeling, osteoblastic cells respond to bacterial stimulation (e.g., to *P. gingivalis* LPS) by expressing cytokines, prostaglandins, and growth factors (e.g., IL‐6, TNF‐α, fibroblast growth factor), all of which mediate inflammation and promote osteoclastogenesis by activating the RANKL‐production signaling pathway [131]. Additionally, the production of inflammatory factors by osteoblasts has been shown to inhibit osteoblastic bone formation by upregulating RANKL production in vivo [132, 133, 134]. Inflammation is responsible for bone resorption by stimulating osteoclastogenesis directly by actions on osteoclasts but also indirectly by limiting bone formation by osteoblasts [135]. For these reasons, osteoblasts are an important therapeutic target to limit alveolar bone resorption.

RvE1 treatment has been shown to significantly decrease the IL‐6‐induced NF‐κB, MAPK, and p53 inflammatory signaling pathways in osteoblasts, which are responsible for multiple osteoblastic functions, including, differentiation, protein synthesis, and apoptosis [136]. In addition, RvE1 has been shown to decrease RANKL production by osteoblasts in a dose‐dependent manner in vitro [137]. Collectively, these results suggest that SPMs can act directly on osteoblasts to produce a microenvironment in favor of bone perseveration and formation.

#### Cell‐cell interactions

**FIGURE 4:**
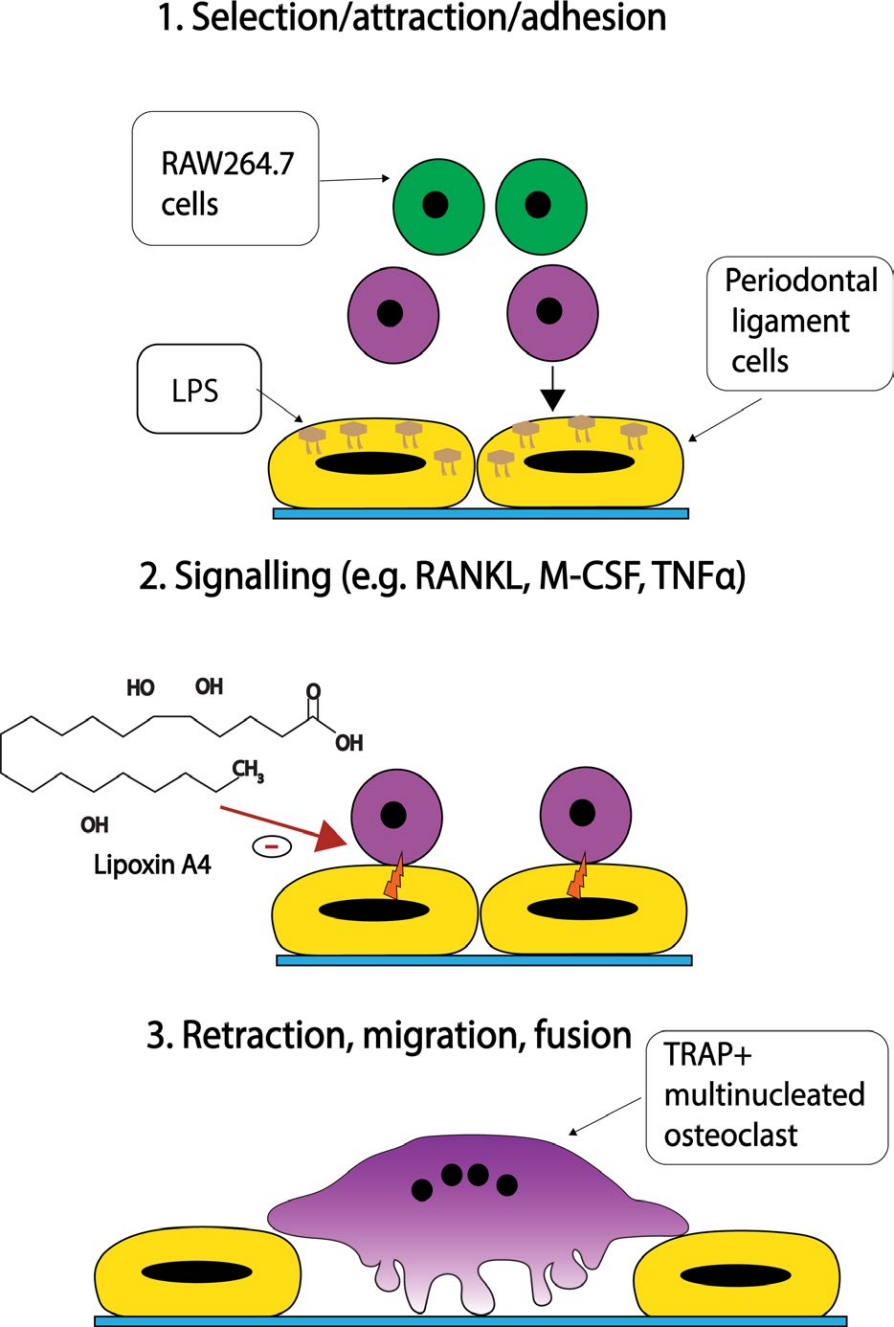
Cell‐cell interactions between osteoclast precursors (OPCs) and osteoclast‐like cells, including periodontal ligament fibroblasts (PDLFs), induce the formation of multinucleated TRAP‐positive osteoclasts in 3 key steps: (1) bacterial lipopolysaccharide (LPS) stimulation induces selection, attraction, and adhesion of OPCs (e.g., RAW264.7 cells), (2) increased inflammatory mediator signaling (e.g, RANKL, M‐CSF and TNFα) promotes the formation of bone‐resorbing osteoclasts, and (3) PDLFs retract and OPCs undergo further maturation into large multinuclear TRAP‐positive osteoclasts.

Cell‐cell contact plays an important role in mediating inflammation‐induced bone resorption due to increased gene expression of adhesion molecules and inflammatory factors, which strongly promote osteoclast differentiation. Cell‐cell interactions between osteoclast precursor cells and osteoblastic cells are crucial for osteoclast differentiation [138] (Figure 4). Contact between osteoclast precursor cells and osteoblastic stromal cells promotes osteoclast differentiation into multinucleated TRAP‐positive osteoclasts. Bone‐lining cells (e.g., PDLFs and gingival fibroblasts) produce high levels of alkaline phosphatase and have been shown to participate in bone formation directly via interactions with osteoclast precursor cells in vitro [139]. Moreover, osteoblasts express osteoclastogenesis‐inducing factors that act in a paracrine manner to influence the proliferation and differentiation of osteoclast precursor cells under inflammatory conditions [140]. In this scenario, RANKL and intracellular adhesion molecule (ICAM‐1) promote osteoclastic differentiation by binding to RANK and leukocyte function associated antigen‐1 receptors, respectively, on osteoclast precursor cells to promote osteoclastic differentiation [141, 142, 143]. Furthermore, osteoblasts produce M‐CSF, which binds to colony‐stimulatory factor 1 (c‐Fms) receptors on osteoclast precursor cells to further promote their differentiation towards the osteoclastic lineage [144]. The expression of pro‐inflammatory molecules (e.g., TNFα, IL‐1β, IL‐6) by osteoblasts can modulate the function of osteoclast precursor cells by affecting the RANKL/osteoprotegerin (OPG) activity [145]. Moreover, pro‐inflammatory cytokines promote RANKL expression and/or decrease OPG expression in osteoclast precursor cells [146, 147]. Ultimately, cell‐cell mediated osteoclastogenesis is required for the differentiation of osteoclast precursor cells into multinucleated osteoclasts and is, therefore, a therapeutic drug target to alleviate inflammation‐induced bone resorption.

Lipoxin (LXA4) has been shown to decrease inflammation‐induced formation of osteoclasts in vitro using murine RAW264.7 in co‐culture with an MC3 T3‐E1 osteoblastic cell line [18]. Li et al. [18] observed that LXA4 inhibited polymethylmethacrylate (PMMA)‐upregulated production of TNFα, IL‐1β, PGE2, and granulocyte‐macrophage colony‐stimulating factor (GM‐CSF) in a dose‐dependent manner. Furthermore, LXA4 counteracted RANKL/LPS‐induced formation and function of osteoclasts in an in vitro co‐culture model composed of primary human PDLFs and murine RAW246.7 cell line [123]. LXA4 suppressed RANKL/LPS‐induced upregulation of osteoclast‐associated genes (e.g., RANKL, TRAP, cathepsin K), TRAP activity, and the number of TRAP‐positive cells. Collectively, these studies suggest that SPMs can be used to inhibit inflammation‐induced bone resorption by minimizing cell‐cell interactions between osteoclast precursor cells and osteoblastic cells.

## CONCLUSION AND FUTURE PERSPECTIVES

The inflammatory response is a protective process designed to eliminate harmful stimuli and promote the resolution of inflammation. In the past, it was generally held that inflammatory resolution was a passive event. However, evidence from studies carried out during the last decades has shown that it is a coordinated active process mediated by a variety of SPMs. Exogenous SPMs, such as lipoxins and resolvins, restore tissue homeostasis by inhibiting leukocyte infiltration and cytokine/chemokine generation. More specifically, SPMs induce apoptosis in PMNs and promote their removal by macrophages. More importantly, exogenous SPMs can be used to reverse inflammation in periodontal and peri‐implant disease and return inflamed tissue to homeostasis. A thorough understanding of the resolution pathways can help to develop a more “personalized” treatment option rather than prescribing one treatment to suit all patients regardless of the causative factor. When viewed in the context of SPMs’ ability to reduce tissue injury in experimental models of inflammation, SPMs can be used to promote resolution pathways and restore tissue homeostasis. A growing body of in vitro and in vivo research suggests modulation of the inflammatory response in several cell types that contribute to the resolution of periodontal inflammation. However, further studies are needed to elucidate the legitimacy of SPMs as a candidate for periodontal therapy. Overall, SPMs warrant further study on the beneficial effect of these agents in periodontal treatment.

## CONFLICT OF INTERESTS

The authors declare that they have no conflict of interest.

## AUTHOR CONTRIBUTIONS

MA contributed to the conceptualization, literature search, writing, editing, and critical review of the article. FY contributed to the project administration, supervision, writing, editing, and critical review of the article. ASP contributed to the writing, editing and critical review of the article. JAJ contributed to the supervision, writing, editing and critical review of the article. XFW contributed to the supervision, reviewing, writing, editing and critical review of the article. All authors approved the final version of this article.
